# Pepsin

**Published:** 1876-11

**Authors:** 


Pepsin.—Dr. Wilkins, in the *Baltimore Physician and Surgeon*, says:

"As experience appears to offer contradictory testimony on the value of pepsin as an aid to artificial digestion, and in consideration of the number of different preparations in the market, it may be a matter of some interest to know the relative value of each, and how far its failure in the hands of certain observers is to be attributed to the quality of the preparation employed. To at least partially meet this indication, at my request, Mr. L. C. Rochle very kindly tested a number of preparations, and gives the following result as the mean average of each: 5 grains of pepsin, mixed with 6 drops of muriatic acid and one ounce of distilled water, dissolved the given quantities of coagulated albumen in six hours, at 100 deg. F.; Sheffer's, 56 grs.; Marson's, 55 grs.; Sharp & Dohme, 54 grs.; French (Boudalt), 54 grs.; Lazell, Marsh & Gardiner, 37 grs.; Hawley's, 13 grs.; Houghton's, 8 grs.

